# Pilot-Scale Production of Hemicellulose Ethers from Softwood Hemicelluloses Obtained from Compression Screw Pressate of a Thermo-Mechanical Pulping Plant

**DOI:** 10.3390/polym15102376

**Published:** 2023-05-19

**Authors:** Petri Widsten, Karl Murton, Tracey Bowers, Jamie Bridson, Armin Thumm, Stefan Hill, Keryn Tutt, Mark West, Garth Weinberg, Gavin Durbin, Christophe Collet

**Affiliations:** Scion, 49 Sala Street, Private Bag 3020, Rotorua 3046, New Zealand; karl.murton@scionresearch.com (K.M.); traceybowers57@gmail.com (T.B.); jamie.bridson@scionresearch.com (J.B.); armin.thumm@scionresearch.com (A.T.); stefan.hill@scionresearch.com (S.H.); keryn.tutt@scionresearch.com (K.T.); mark.west@scionresearch.com (M.W.); garth.weinberg@scionresearch.com (G.W.); gavin.durbin@scionresearch.com (G.D.); christophe.collet@scionresearch.com (C.C.)

**Keywords:** hemicellulose, effluent, ether, modification, radiata pine, thermo-mechanical pulping, ultrafiltration

## Abstract

Bio-derived materials are becoming increasingly sought-after as a sustainable alternative to petrochemical-derived polymers. In the present pilot-scale study, a hemicellulose-rich compression screw pressate, obtained from the pre-heating stage of thermo-mechanical pulping (TMP) of radiata pine, was purified by treatment with adsorbent resin (XAD7), then ultrafiltered and diafiltered at 10 kDa to isolate the high-molecular weight (MW) hemicellulose fraction (yield 18.4% on pressate solids), and, finally, reacted with butyl glycidyl ether to plasticise the hemicelluloses. The resulting light brown hemicellulose ethers (yield 102% on the isolated hemicelluloses) contained ca. 0.5 butoxy-hydroxypropyl side chains per pyranose unit and had weight- and number-average MWs of 13,000 Da and 7200 Da, respectively. The hemicellulose ethers may serve as raw material for bio-based products such as barrier films.

## 1. Introduction

Hemicelluloses are amorphous polysaccharides such as O-acetyl-galactoglucomannan (GGM) and O-acetyl-(4-O-methylglucurono)-β-D-xylan (xylan) that are the main types of hemicellulose in softwood and hardwood cell walls, respectively. Their total content typically ranges from 15 to 30% of dry wood matter [1,2]. According to Smelstorius [3], the wood of radiata pine (*Pinus radiata*) contains 17.5% GGM and 9.2% arabinoxylan as well as 5.0% other non-cellulosic polysaccharides (arabinogalactan, pectic substances and arabinan). The MW of GGM is about 16,000 to 24,000 Da [4].

Barrier films made from materials derived from lignocellulosic feedstock such as wood hemicelluloses would constitute an environmentally sustainable alternative to commercial plastic barrier films manufactured from petrochemicals. Xylan and GGM extracted from bleached kraft pulps have previously been etherified with epoxy-functional ethers (propylene oxide, allyl glycidyl ether and/or butyl glycidyl ether) to plasticise them and render them suitable for coatings, films or hydrogels [5,6,7,8,9]. The partial etherification of hemicellulose hydroxyl groups yielded xylan and GGM ethers differing in their hydrophobicity, side-chain length and structure, and degree of substitution. In one study, xylan isolated from bleached kraft pulp was plasticised by hydroxypropylation and cast into films showing good oxygen barrier properties [6] that are relevant, e.g., for food packaging. However, kraft pulp is a relatively expensive source of hemicellulose for film-making and it would be better to use hemicelluloses isolated from by-products of the forest products industry such as thermo-mechanical pulping (TMP) and fibreboard pressing effluents [10,11].

A laboratory-scale process for the extraction and purification of radiata pine high-MW hemicelluloses (mainly GGM) from a compression screw pressate (CSP) of a TMP plant using different severity factors in the pre-heater was published recently [12]. The severity factor affected the yield and composition of the pressate solids and the optimal combination of pre-heating temperature and residence time was ascertained. The present follow-up investigation describes a pilot-scale (600 kg) process for the isolation, purification and etherification with butyl glycidyl ether (BGE) of plasticised CSP-derived high-MW hemicelluloses that may be suitable for barrier film applications.

## 2. Materials and Methods

### 2.1. Preparation of Compression Screw Pressate (CSP)

Compression screw pressate (CSP) ‘160/20’ from freshly debarked pine (*Pinus radiata* D. Don) slabwood chips from a sawmill located in Rotorua, New Zealand, was produced in the pre-heater of a pilot-scale TMP plant as described earlier [12] and stored in the dark at 4 °C until needed. The temperature (160 °C) and duration (20 min) of the pre-heating stage were those that, in a previous study, gave a CSP with the highest content of high-MW hemicellulose [12]. For a detailed process diagram, see Lloyd and Murton [10].

### 2.2. Determination of Total Dissolved Solids (TDS)

Total dissolved solids (TDS) in filtered 160/20 samples were determined gravimetrically after freeze-drying 100 g samples.

### 2.3. Isolation of the High-MW Hemicellulose Fraction

The 160/20 (ca. 600 kg) was filtered through a Nybolt 75/36 nylon mesh (pore size 75 μm; purchased from Clear Edge Filtrations, Auckland, New Zealand) to remove fines and then treated with XAD7 resin (Chemsphere Technology Inc., Taipei, Taiwan) to reduce the amount of non-hemicellulose material such as pseudo-lignin/humin [13,14,15] or depolymerised lignin [16] that has a negative impact on the flow rate [17]. Before use, the resin was washed with deionised water to remove the preservative salts then recovered by filtration through nylon cloth and oven-dried at 50 °C. The 160/20 (ca. 590 kg) was treated with the washed XAD7 in 2 batches for 3 h at room temperature in a 500 L baffled steel drum equipped with a vortex mixer at a resin loading of 8% (based on washed resin weight on 160/20 solids). The resin was then regenerated by washing it with 0.1 M NaOH and deionised water until the washings were nearly colourless and the washed resin was used to repeat the treatment with the virgin resin. The purified CSP, ‘160/20-XAD7’ (550 kg), was then ultrafiltered (UF) and the UF retentate (116 kg) diafiltered (DF) twice at FoodPilot (Massey University, Palmerston North, New Zealand) at a membrane cut-off of 10,000 Da. Prior to the two diafiltrations, the volume of permeate removed during the UF step was added to the previous retentate. The final DF retentate, ‘R3’ (90 kg), was kept frozen at −30 °C until needed. The details of the UF/DF process were as follows:

The UF plant was set up with 2 10 kDa cut-off membranes (GE Osmonics serial numbers 65293 and 69292). The plant was cleaned and sanitised according to standard operating procedures. Cleaning and sanitising were carried out before and after each run.

A total of 493 kg of sample was passed through the UF plant and concentrated until the final volume was approximately 100 L (a tankful). The feed tank temperature started off at 3.2 °C and gradually increased to 22 °C by the end of the day. The permeate flow rate started off at 3.13 L/min and gradually decreased to 0.63 L/min. The permeate Brix reading increased from 1.6 to 3.1. The retentate flow rate started at 4.2 L/min and slowly increased to 6.6 L/min. The initial Brix reading was 4.9, increasing to 14.4. This took a total of 550 min over 2 days to achieve. The amount of permeate collected was 435 L which was mixed in the modicon. A 100 mL sample and 6 5 L samples were taken and frozen and the remainder of the permeate was discarded. The Brix reading of the combined permeate was 2.5. The valve at the bottom of the feed tank was closed and the membranes drained of all retentate, and this was added back to the feed tank to ensure uniform mixing of the sample, as a concentration gradient may be set up between feed tank and membrane loop. A 100 mL sample was taken and frozen. The retentate Brix reading was 14.4.

The first retentate (R1) was then diafiltered. The feed tank was kept at the same level by continuously adding soft water (until the feed tank overflowed) and the volume of permeate collected was monitored. Diafiltering was continued until the same amount of permeate (435 kg) that was collected in the ultrafiltration was achieved. This took 639 min over 2 days. The permeate flow rate averaged about 0.6 L/min on the first day (489 min) and was about 1 L/min on the second day. The retentate flow rate started above 6 L/min, and finished between 5 and 6 L/min. The feeder tank temperature started at 20 °C and finished at 23 °C on the first day, and started at 13.8 °C on the second day, finishing at 20 °C. The permeate was mixed in the modicon and a 100 mL sample was taken and stored frozen, and the remainder was discarded. The Brix reading of the combined permeate was 1.0. The retentate (R2) was mixed as above and had a Brix reading of 9.2. A 100 mL sample was taken and stored frozen and the retentate was further diafiltered for a second time.

The diafiltration of R2 was carried in the same way and took 352 min to complete over 1 day. The permeate flow rate averaged 1.26 L/min, and the retentate flow rate averaged 5.43 L/min. The feeder tank temperature started at 20 °C and finished at 20.5 °C. The permeate was mixed in the modicon and was found to have a Brix reading of 0. A 100 mL sample was taken and frozen and the remainder was discarded. The retentate was further ultrafiltered until another 40 kg of permeate had been removed to reduce the overall volume and to ensure the solids were as high as possible. The final Brix reading was 9.8. The final retentate (R3) was drained from the membranes and added back to the feed tank for mixing as described above and a 100 mL sample taken. The remainder was transferred to 18 10 L containers (5 L per container) and stored frozen.

### 2.4. Measurement of UV Absorption

The UV absorbance of CSP samples was measured at 280 nm in a quartz cell with a path length of 1 cm using a Shimazdu UV-1800 UV-Vis spectrophotometer. Before measurement, samples were diluted with Milli-Q water until the absorbance readings were in the range 0.2–1.0. The measurements were performed in triplicate and the results expressed as the values obtained by multiplying the readings with the corresponding dilution factors.

### 2.5. Determination of Wood Extractives

Quantification of wood extractives in CSP samples was performed in triplicate using an earlier published method [12,18,19].

### 2.6. Determination of Hemicellulose MW Distribution

Gel permeation chromatography (GPC) was used to determine the MW distribution of freeze-dried CSP samples as described in [12].

### 2.7. Determination of Klason and Acid-Soluble ‘lignin’ Contents

Acid-insoluble Klason ‘lignin’ in freeze-dried pressate was determined by a modified method based on TAPPI Standard Methods, T 222 om-88 and acid-soluble ‘lignin’ by a modified TAPPI Useful Method UM 250. Determinations were performed in triplicate and the mean value reported. It should be noted that these ‘lignins’ may not originate from native lignin but in fact be pseudo-lignin/humin of hemicellulose origin [13].

### 2.8. Determination of Volatile Organic Compounds (VOCs)

Volatile fatty acid concentrations (C2–C6) were determined by an in-house method involving pH correction with formic acid followed by capillary gas chromatography with flame ionisation detection (GC-FID) using an Agilent 7890 GC unit equipped with a nitroterephthalic acid modified polyethylene glycol capillary column, DB-FFAP: 30 m × 530 μm × 0.5 μm ramped from 40 °C to 180 °C. Butan-1-ol solution served as an internal standard, and turbid samples were pre-filtered through a nylon syringe filter (0.45 μm). The method was also used to determine low-MW alcohol (C1–C6) concentrations.

### 2.9. Determination of Furans

Samples in tetrahydrofuran (THF, ≥99.9%, Sigma-Aldrich, St. Louis, MO, USA) were diluted with water 1 in 50, filtered through a 0.2 μm membrane and analysed by high-performance liquid chromatography (HPLC). HPLC was carried out on an Agilent 1290 system with diode array detection, using a YMC UltraHT Pro C18 100 mm × 2.1 mm I.D. column with 2 μm particle size and 12 nm pore. The mobile phases were A: water + 0.1% trifluoroacetic acid (TFA, ≥99.0, Sigma-Aldrich, St. Louis, MO, USA) and B: acetonitrile (≥99.9%, Sigma-Aldrich, St. Louis, MO, USA) + 0.1% TFA. A gradient from 20–60% B over 10 min followed by a 1.5-min re-equilibration to 20% B was used to separate the components at a flow rate of 0.2 mL/min. Detection was at 220 nm (furfuryl alcohol), 260 nm (2-furoic acid) and 280 nm (furfuraldehyde, 5-hydroxymethylfurfural, 5-methylfurfural). The injection volume was 1.0 μL. Analytical standards of furfuryl alcohol, furfuraldehyde, 2-furoic acid, 5-hydroxymethylfurfural and 5-methylfurfural from Sigma-Aldrich (St. Louis, MO, USA) were used to identify and quantify compounds.

### 2.10. Solid-State ^13^C CP/MAS NMR Spectroscopy

Samples (enough freeze-dried material to fill a 156 μL volume) analysed by solid-state ^13^C nuclear magnetic resonance (NMR) spectroscopy were spun at 5 kHz in a 4 mm Bruker (Faellanden, Switzerland) SB magic angle spinning (MAS) probe at 50.3 MHz using a Bruker (Faellanden, Switzerland) Avance III 200 spectrometer. Cross-polarisation (CP) experiments consisted of a 1.5 s pulse delay followed by a 4.25 ms proton preparation pulse, a 1 ms contact time and a 26 ms acquisition time with high-powered proton decoupling. All spectra had a Gaussian line broadening of 25 Hz applied prior to Fourier transform and were calibrated so that the cellulose interior C4 peak was assigned a value of 89.3 ppm, previously established relative to polydimethylsilane at −1.96 ppm, in turn measured relative to tetramethylsilane at 0 ppm.

### 2.11. FTIR Spectroscopy

Fourier-transformation infrared (FTIR) spectra of freeze-dried samples were run in the ATR (attenuated total reflectance) mode on a Bruker (Faellanden, Switzerland) Optics Tensor 27 FTIR spectrometer. The spectra (4000–800 cm^−1^, 16 scans) were recorded in the absorbance mode then baseline corrected and normalised to the carbohydrate band at ca. 1032 cm^−1^ using OPUS version 7.2 software.

### 2.12. Synthesis and Purification of 3-Butoxy-2-Hydroxypropylated Hemicellulose

**Figure 1:**
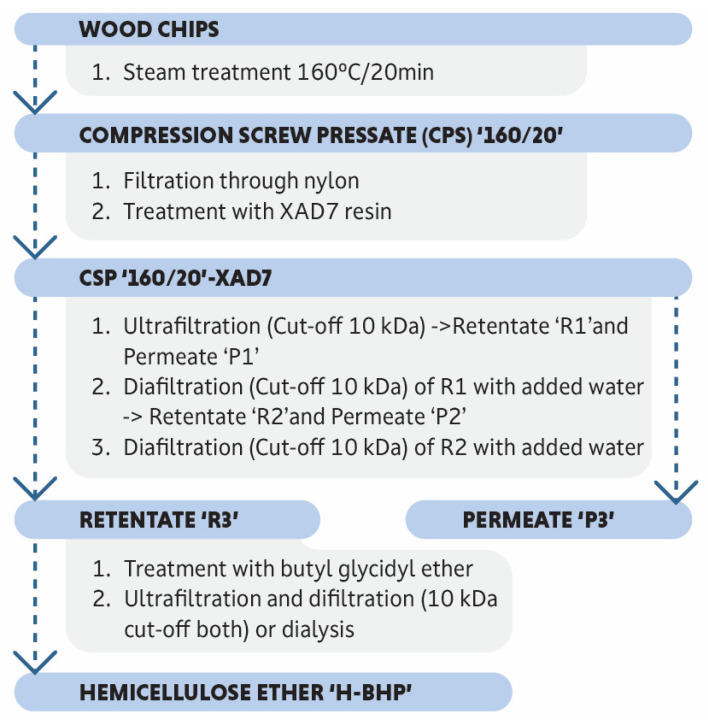
Schematic description of the manufacturing process of hemicellulose ethers.

Hemicellulose 3-butoxy-2-hydroxypropyl ether was produced in a 100 L stainless steel reactor (Biostat DDCU, Sartorius, Germany) equipped with a heating jacket, 3 Rushton impellers at different heights, pH probe, dissolved oxygen probe and inlets for the reagent and nitrogen gas. Hemicellulose solution (Retentate 3; 45 kg; 9.5% solids) and enough 10 M NaOH to provide a 1 M NaOH final concentration were placed in the reactor and stirred at 300 rpm for 1 h at 45 °C after which the solution was purged by nitrogen sparging. Then, 2 molar equivalents (based on the molar mass of a pyranose unit of 162 g/mol) of the etherification reagent, butyl glycidyl ether (BGE, supplied by Shanghai Terppon Chemical Co. Ltd., Shanghai, China), were added by injection through a septum in batches of 30 mL over 1 h while stirring (300 rpm) and the temperature was maintained at 45 °C. Antifoaming agent (0.7 mL; poly(propylene glycol)-block-poly(ethylene glycol)-block-poly(propylene glycol), Merck, Darmstadt, Germany) was also added. The reaction was allowed to run for 16 h under these conditions after which the solution was cooled to 30 °C and neutralised to pH 7.6 by slow addition of 20% sulphuric acid. The neutralised solution was purified by dialysis (Spectra/Por 1, Spectrum Laboratories, Inc., Rancho Dominguez, US; cut-off of 6000–8000 Da) until the conductivity was <1 mS/cm. The purified product, 3-butoxy-2-hydroxypropylated hemicellulose (H-BHP), was then freeze-dried. A schematic representation of the process from wood chips to H-BHP is shown in Figure 1.

## 3. Results and Discussion

### 3.1. Characterisation of CSP 160/20

**Figure 2:**
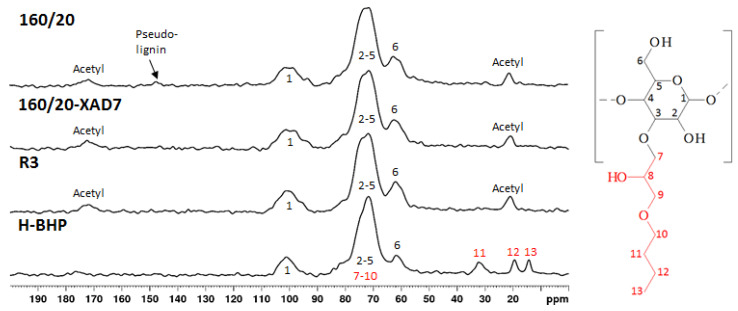
Solid-state ^13^C CP/MAS NMR spectra of freeze-dried CSP and hemicellulose ether (H-BHP) samples.

**Figure 3:**
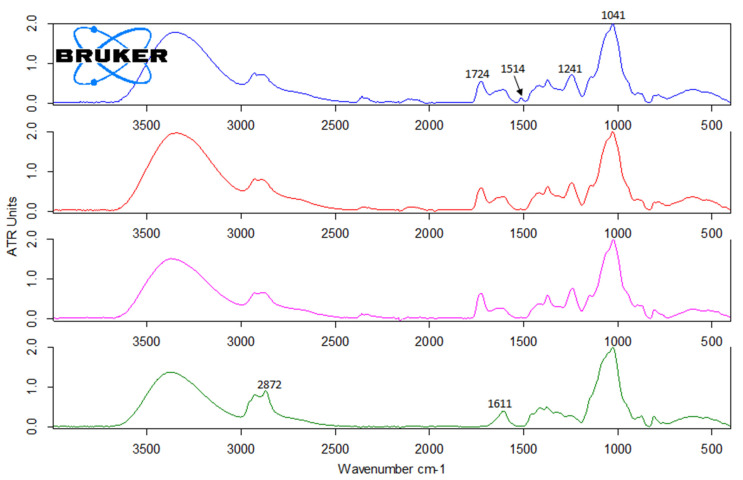
FTIR-ATR spectra of freeze-dried CSP samples. From **top** to **bottom**: 160/20; 160/20-XAD7; R3; H-BHP.

**Figure 4:**
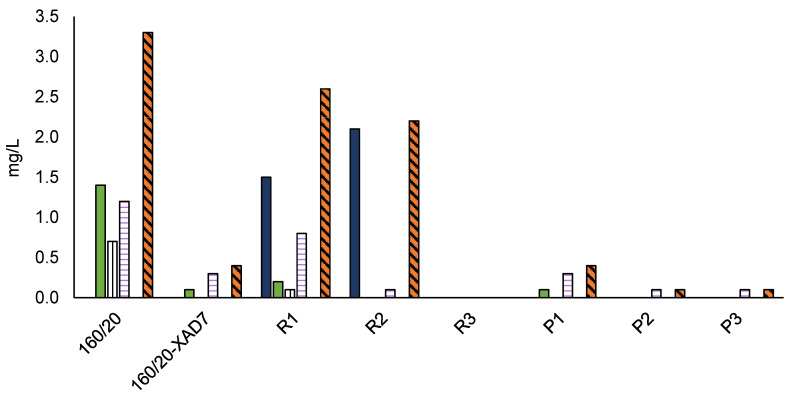
Furanic hemicellulose degradation products detected in CSP samples.

**Figure 5:**
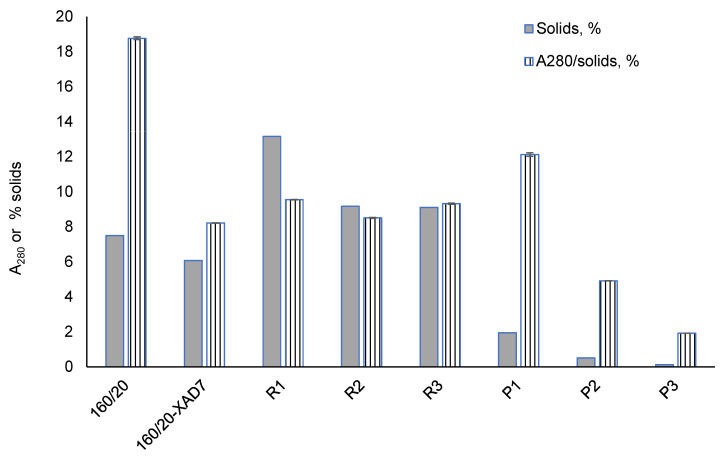
Solids content and ratio of UV absorption at 280 nm to solids content of CSP samples. Error bars show standard deviation based on absorption measurements.

An earlier laboratory-scale investigation [12] showed that CSP 160/20 contains ca. 91% mono- and oligosaccharides, reflecting the known non-cellulosic polysaccharides in radiata pine wood (galactoglucomannan, xylan, arabinoxylans and arabinogalactans [3] and ca. 8% of aromatic material, potentially comprising lignin, pseudo-lignin and monomeric furans. In the present study, a more thorough characterisation of the non-saccharide components was carried out. The solid-state ^13^C NMR spectrum of 160/20 (Figure 2) shows peaks assigned to hemicellulose saccharides (pyranose units and acetyl) [20] and one peak centred at 148 ppm attributed to aromatic pseudo-lignin (humin) impurities [13,14,15]. The peak assignments are corroborated by the FTIR spectrum of 160/20 (Figure 3) showing a strong band at 1721 cm^−1^ (acetyl groups in hemicellulose) and a small but well-defined band at 1514 cm^−1^ (aromatic C=C stretching in pseudo-lignin/humin) [13]. As pseudo-lignin/humin has also been reported to produce bands at 1697 cm^−1^ (C=O stretching) and 1611 cm^−1^ (aromatic C=C stretching) [13], it may contribute to the poorly defined bands in the region 1600–1700 cm^−1^. It is formed via polymerisation or condensation of the initial furanic degradation products of hemicellulose such as 5-hydroxymethylfuran (HMF) and furfural, which were also identified in trace amounts in 160/20 (Figure 4), and monosaccharides [14,15]. The contribution of depolymerised lignin to peaks arising from pseudo-lignin/humin is probably small because the pressate was obtained from wood chips that were not mechanically refined (defibrated). As pseudo-lignin/humin is largely aromatic and thus absorbs UV light, UV absorption can be used without calibration with model compounds to estimate the relative amounts of pseudo-lignin and low-MW furans in different samples. The strong absorbance of 160/20 in the UV region at 280 nm (Figure 5) is thus in line with the spectroscopy results.

**Figure 6:**
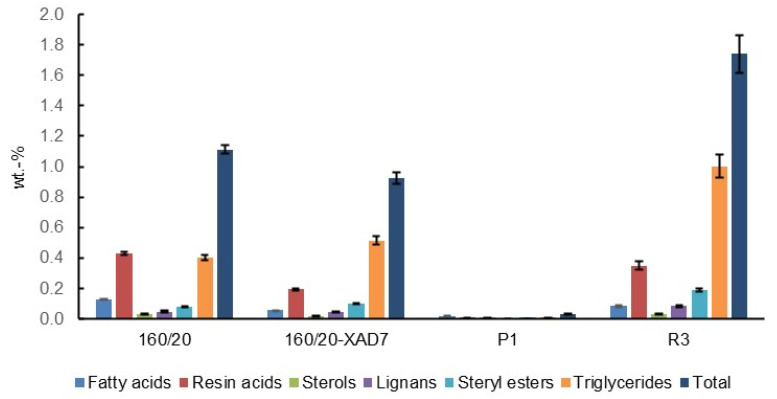
Lipophilic extractive content of the total solids in selected CSP samples. Error bars show standard deviation.

**Figure 7:**
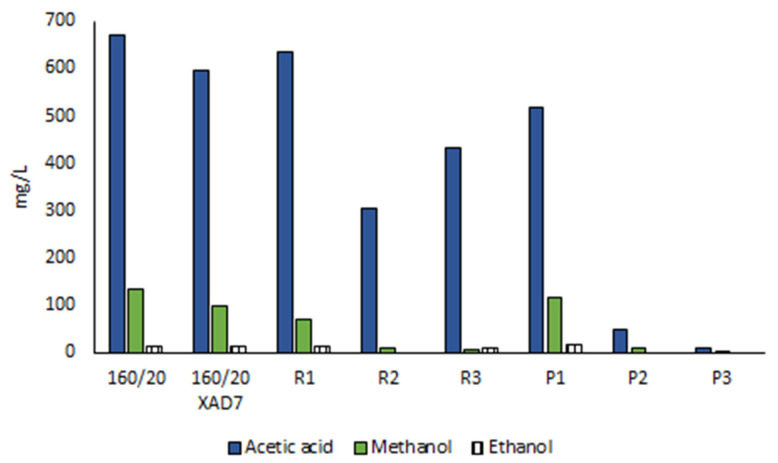
The three most prominent VOC compounds in CSP samples (mean values of duplicate determinations).

Other compounds detected in 160/20 include lipophilic wood extractives (Figure 6) and volatile organic compounds (VOCs; Figure 7). The lipophilic extractives contributed approximately 1% of the CSP total solids content, consisting primarily of resin acids and triglycerides, while the VOC component comprised mainly acetic acid and methanol. Acetic acid is formed by deacetylation of O-acetyl-galactoglucomannan [21] while demethylation of arabino-4-O-methylglucuronoxylan can account for the methanol. Traces of ethanol and linear C3-C6 organic acids were also detected.

### 3.2. Effect of XAD7 Resin Treatment on 160/20

**Figure 8:**
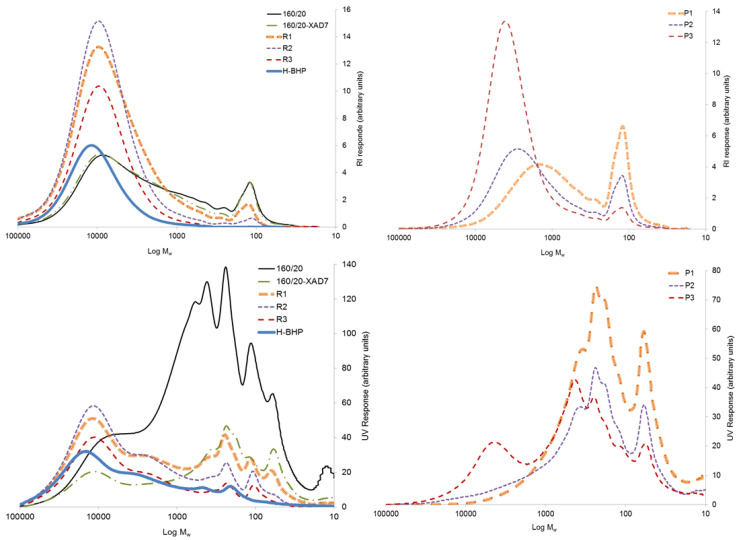
GPC MW traces of freeze-dried samples obtained with RI detector (**top**) and UV detector (**bottom**).

**Figure 9:**
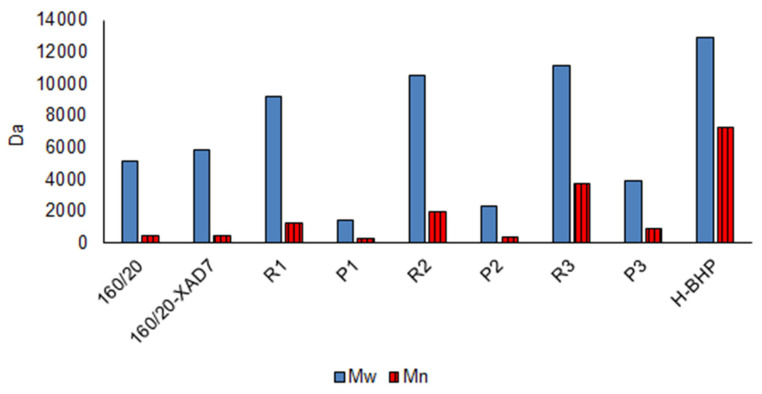
Molecular weight distribution of freeze-dried samples (RI detector).

The 160/20 was treated with XAD7 resin to reduce the amount of non-hemicellulose material (pseudo-lignin/humin, low-MW furans and wood extractives) prior to ultrafiltration. Direct ultrafiltration was not possible as the membrane fouling by these non-sugar low-MW contaminants immediately reduced flux to minimal. The treatment reduced the solids content by 19%. A comparison of 160/20 and 160/20-XAD7 shows a 56% reduction of UV absorption at 280 nm adjusted for solids concentration (Figure 5) while the small but distinct peaks assigned to aromatic pseudo-lignin/humin in the NMR (145–150 ppm) and FTIR (1513 cm^−1^) spectra were practically eliminated (Figure 2 and Figure 3). There was also an 88% reduction in the concentration of strongly UV-absorbing low-MW furans (Figure 4), whose absorption onto XAD resins has been reported [22]. In addition, the GPC traces obtained with a UV detector attest to a large decrease in UV-absorbing impurities (Figure 8). Regarding other impurities, the content of lipophilic wood extractives decreased by 17%, the smaller extractive molecules (free fatty and resin acids; sterols) being responsible for this reduction (Figure 6), while VOCs declined by 11% (Figure 7). Furthermore, as seen from the GPC chromatographs obtained with the universal RI detector (Figure 8) and the calculated MW distributions (Figure 9), the overall MW distribution did not change significantly. These results show that the resin treatment was effective at removing UV-absorbing impurities without losing valuable high-MW polysaccharides.

### 3.3. Ultra- and Diafiltration of 160/20-XAD7

**Figure 10:**
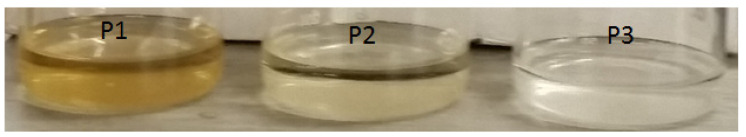
Samples of UF permeate P1 and DF permeates P2 and P3.

The 160/20-XAD7 was ultrafiltered at a membrane cut-off of 10,000 Da and the high-MW saccharides in the retentate further purified by double DF (before a DF stage, the volume of permeate removed during the previous stage was replaced with clean water). The NMR and FTIR spectra of 160/20-XAD7 and the final DF retentate (R3) are very similar (Figure 2 and Figure 3). When adjusted for solids content, the UV absorption values of the three retentates R1-R3 were like those of 160/20-XAD7 (Figure 5). Based on the UV absorptions of the three permeates P1-P3, adjusted for solids, most of the impurities removed passed into the UF permeate P1 that was also more strongly coloured than the DF permeates P2 and P3 (Figure 10).

Low-MW furans were present in all the retentates and permeates, albeit in trace amounts (Figure 4). The GPC UV traces show that the low-MW UV-absorbing impurities removed were mostly of very low-MW (<1000 Da) while the impurities remaining in the final retentate (R3) were mostly of high-MW (Figure 8). The lipophilic wood extractive levels were higher in R3 than in 160/20-XAD7, with resin acid and triglyceride levels approximately doubling (Figure 6). As for VOCs (Figure 7), their content of methanol was progressively reduced in the retentates (going from R1 to R3) while most of the acetic acid in 160/20 was carried over to R3 (and/or further deacetylation of GGM took place during the process). However, a comparison of the NMR and FTIR spectra of 160/20 and R3 shows that the extent of deacetylation was not significant (Figure 2 and Figure 3).

The weight-average molecular weight (M_w_) of the retentate saccharides increased progressively from R1 to R3 (Figure 9). Permeates P1-P3 showed the same trend while their solids content decreased, hence the smallest molecules tended to be filtered out sooner.

Overall, the UF/DF process was effective at concentrating the high-MW saccharides in 160/20-XAD7 to R3, but the purity in terms of the ratio of saccharides to the content of UV-absorbing compounds or lipophilic extractives was not improved. The high-MW compounds in R3 may have been pseudo-lignin and/or lignin attached to carbohydrates (lignin-carbohydrate complexes, LCCs). The yield of R3 solids on 160/20 solids was 18.4%.

### 3.4. 3-Butoxy-2-Hydroxypropylation of High-MW Hemicellulose (R3)

**Figure 11:**
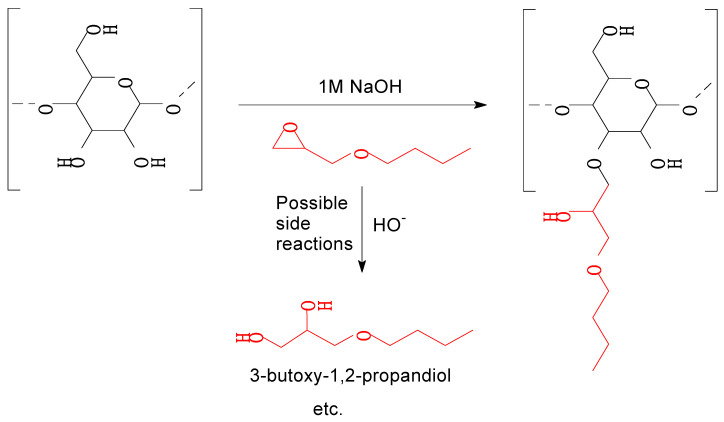
Synthesis of 3-butoxy-2-hydroxypropylated hemicellulose. The site(s) of reaction may be any one or more of the three pyranose hydroxyl groups (reaction at a single hydroxyl is shown here as an example). Hydrolytic side reactions such as the one depicted above are also possible. The reactions are expected to proceed mostly through backside S_N_2 attacks on the epoxy ring at its less hindered carbon.

High-MW hemicellulose R3 was reacted with BGE (see Figure 11) to plasticise it and the product neutralised and purified by dialysis. The aim of the dialysis was to eliminate salts, any unreacted BGE or its hydrolysis products (such as 3-butoxy-1,2-propandiol, formed via ‘backside’ S_N_2 reaction of hydroxyl anion at the less sterically hindered carbon of the epoxy ring; Figure 11) and etherified low-MW saccharides (<6000 Da) not considered by the authors suitable for film-making. The unpleasant odour of the neutralised reaction mixture indicated the presence of BGE and/or its hydrolysis products. The yield of the freeze-dried purified product (H-BHP) was 4.36 kg (102% on R3 solids). The combined effects of the 3-butoxy-2-hydroxypropyl side chains introduced, and the removal of residual small molecules, resulted in substantial increases in MW, especially in M_n_ (Figure 9). New peaks, assigned to the methyl and methylene carbons not directly bonded to oxygen in the 3-butoxy-2-hydroxypropyl chain, appeared in the quantitative CP/MAS solid-state ^13^C NMR spectrum of the product (Figure 2). Its degree of substitution (no. of etherified pyranose hydroxyls per pyranose unit) was calculated as 0.5 based on the butyl methylene and methyl peaks and the pyranose anomeric C1 peak, similar to those obtained for xylan under similar reaction conditions [5]. The acetyl peaks from hemicellulose were eliminated, indicating that acetyl groups were fully hydrolysed in the course of the reaction, which was carried out at highly alkaline conditions. The FTIR spectrum of H-BHP (Figure 3) agrees with the NMR spectrum: the acetyl peak has disappeared while changes are seen in the area between 800 and 1300 cm^−1^ containing signals from C-O stretching in alcohols and ethers. The increase in the band centred at 2872 cm^−1^ may be due to the methyl group of the ether side chain. The area where carbonyl structures from impurities would be seen from 1500 to 1700 cm^−1^ now contains only a well-defined peak centred at 1611 cm^−1^, assigned to residual pseudo-lignin impurities and/or absorbed moisture. The GPC UV trace (Figure 8) shows a decline in residual UV-absorbing impurities during dialysis—those that still remained included large-size (pseudo-lignin) molecules and had an M_w_ of ca. 8500 Da.

In summary, this pilot-scale process yielded partially purified high-MW hemicelluloses at a yield of 18.4% on starting pressate solids. The remaining impurities (pseudo-lignin/humin of mostly high MW) that absorb light in the UV and visible wavelength regions are responsible for their brownish colour. Etherification of the high-MW hemicelluloses with BGE and dialysis of the reaction mixture produced brownish hemicellulose ethers (M_w_ 13 kDa) at a yield of 102%. If the current degree of substitution turns out to be insufficient to impart enough plasticity to the ethers, e.g., for bio-based barrier films, it is possible to increase it by applying a higher dose of BGE on hemicellulose. Treatments to increase the brightness of the hemicellulose ethers could also be integrated to the process. Finding applications for the mono- and oligosaccharides removed at the UF/DF stage would improve the commercial feasibility of the overall process.

## 4. Conclusions

- High-MW hemicelluloses were isolated from TMP compression screw pressate by XAD7 adsorbent resin treatment (purification) and a UF/DF process (concentration). Purity was significantly improved during the initial treatment with XAD7 resin but not during the subsequent UF/DF process that concentrated the high-MW fractions of both hemicelluloses and UV absorbing impurities.
- The high-MW hemicelluloses (yield 18.4%) were plasticised with butyl glycidyl ether, giving hemicellulose ethers (M_w_ 13 kDa, yield 102% after dialysis of the reaction mixture). The reaction added approximately 0.5 3-butoxy-2-hydroxypropyl side chains per pyranose unit.
- Future work may involve further purification of the high-MW hemicelluloses using oxygen-based bleaching chemicals and ascertaining the potential of hemicellulose ethers to form barrier films.

## Data Availability

Data will be made available on request.
